# Intranasal oxytocin increases heart-rate variability in men at clinical high risk for psychosis: a proof-of-concept study

**DOI:** 10.1038/s41398-020-00890-7

**Published:** 2020-07-12

**Authors:** Daniel Martins, Cathy Davies, Andrea De Micheli, Dominic Oliver, Alicja Krawczun-Rygmaczewska, Paolo Fusar-Poli, Yannis Paloyelis

**Affiliations:** 1 Department of Neuroimaging, Institute of Psychiatry, Psychology and Neuroscience, King’s College London, De Crespigny Park, London, SE5 8AF UK; 2 Early Psychosis: Interventions & Clinical detection (EPIC) lab, Department of Psychosis Studies, Institute of Psychiatry, Psychology and Neuroscience, King’s College London, London, SE5 8AF UK; 3 OASIS Service, South London and the Maudsley NHS National Health Service Foundation Trust, London, UK; 4 Department of Brain and Behavioural Sciences, University of Pavia, Pavia, Italy

**Keywords:** Psychiatric disorders, Pharmacology

## Abstract

Autonomic nervous system (ANS) dysfunction (i.e., increased sympathetic and/or decreased parasympathetic activity) has been proposed to contribute to psychosis vulnerability. Yet, we still lack directed therapeutic strategies that improve ANS regulation in psychosis or at-risk states. The oxytocin system constitutes a potential therapeutic target, given its role in ANS regulation. However, whether intranasal oxytocin ameliorates autonomic regulation during emerging psychosis is currently unknown. We pooled together two datasets, one of 30 men at clinical high risk for psychosis (CHR-P), and another of 17 healthy men, who had participated in two double-blinded, placebo-controlled, randomised, crossover MRI studies with similar protocols. All participants self-administered 40 IU of intranasal oxytocin or placebo using a nasal spray. We recorded pulse plethysmography during a period of 8 min at about 1 h post dosing and estimated heart rate (HR) and high-frequency HR variability (HF-HRV), an index of cardio-parasympathetic activity. CHR-P and healthy men did not differ at resting HR or HF-HRV under placebo. We found a significant condition × treatment effect for HF-HRV, showing that intranasal oxytocin, compared with placebo, increased HF-HRV in CHR-P but not in healthy men. The main effects of treatment and condition were not significant. In this proof-of-concept study, we show that intranasal oxytocin increases cardio-parasympathetic activity in CHR-P men, highlighting its therapeutic potential to improve autonomic regulation in this clinical group. Our findings support the need for further research on the preventive and therapeutic potential of intranasal oxytocin during emerging psychosis, where we lack effective treatments.

## Introduction

Psychotic disorders are among the world’s leading causes of disability^1^. Psychosis is often preceded by subtle features, allowing early detection and prevention^2^. Preventive approaches in psychosis are grounded on the detection^3^, prognostic assessment^4^ and treatment^5^ of individuals at clinical high-risk for psychosis (CHR-P). CHR-P individuals accumulate risk factors for psychosis^6–8^ that lead to attenuated positive psychotic symptoms^9^, impaired functioning^10^ and help-seeking^11^. These individuals have approximately 22% risk of developing a first-episode psychosis over the following 3 years^12^. Currently, there is no effective intervention that can impact on transition to psychosis, symptom severity or social/functional outcomes in CHR-P^5^. Therefore, novel treatments for this population are urgently needed^5^.

Impaired autonomic nervous system (ANS) response (i.e., increased sympathetic and/or decreased parasympathetic activation) to environmental challenges has been proposed as a link between the everyday experience of stressors and the emergence of psychotic symptoms—a core pathophysiological mechanism highlighted in stress–diathesis models of psychosis onset^13,14^. Exposure to psychosocial stress, such as life events, childhood trauma or discriminatory experiences in highly vulnerable individuals, is thought to progressively increase the behavioural and biological (ANS and stress endocrine axis (HPA axis)^15^) responses to subsequent exposures (a processed one called behavioural sensitisation)^15–17^. Ultimately, this heightened response to enduring stressors might contribute to sensitise the dopaminergic pathways^18,19^, which have been proposed to underlie psychosis onset^15^.

Supporting the contribution of heightened stress response to psychosis, previous studies have reported ANS dysfunction during established psychosis and in CHR-P individuals, beyond what might be explained by medication alone^20^. Whether this increased sensitivity to stress is due to a lack of cognitive resources in these patients, such as hampered coping skills or cognitive impairment, or due to the inadequate response of the biologic systems involved in the stress response, is to be fully elucidated yet. Besides, ANS dysfunction has also been suggested to contribute to the heightened cardiovascular risk that accompanies these disorders^21,22^. The psychotherapeutic interventions indicated for CHR-P individuals do not specifically address ANS dysfunction^23^. Therefore, developing new therapies to improve ANS regulation in psychosis and CHR-P individuals might hold promise to prevent transition into full-blown psychosis and/or relapse and address potential cardiovascular comorbidities^2^.

Heart rate (HR), the beat-to-beat fluctuation of instantaneous heart period over time and HR variability (HRV), provide inexpensive and non-invasive proximal measures of the ANS activity^24^. Both the sympathetic and the parasympathetic branches of the ANS dually innervate the heart and modulate the heart rhythm^25^. Sympathetic activity accelerates HR and decreases HRV, whereas parasympathetic activity has the opposite effect. Therefore, the inspection of HR and its resulting variability allows to draw inferences on the efferent activity of the ANS^24^.

Previous studies have demonstrated increases in HR^26–28^ and decreases in HRV during rest in patients with schizophrenia compared with healthy control groups^20^. Similar patterns have been found in first-degree relatives^29–31^ and in CHR-P individuals^32,33^. The definitive mechanisms by which cardiac ANS regulation might be disrupted, leading to the increases in HR and decreases in HRV observed in emerging psychosis, are currently unknown. Consequently, we still lack targeted interventions to improve cardiac ANS regulation during emerging psychosis.

Oxytocin has been considered a promising compound for treating both positive and negative symptoms of psychosis^34–36^. Oxytocin participates in the modulation of several social and cognitive processes that are likely to contribute to the generation of both positive and negative symptoms (e.g., salience, reward processing and social approach). Intranasal oxytocin does not produce significant side effects, is highly tolerable and is not associated with adverse outcomes when delivered in the 18–40-IU doses typically used in single acute administration human studies^37^ (including in children^38,39^). Moreover, two small studies in patients with obsessive compulsive disorder have shown that daily doses of 160–320 IU do not increase the risk of side effects and are well tolerated^40,41^. Therefore, in contrast to other drugs, intranasal oxytocin could hold therapeutic potential for improving positive and negative psychosis symptoms with minimal side effects. Evidence to favour specific preventive treatments over each other for improving positive or negative symptoms in CHR-P is currently limited^5,42,43^. Some intranasal oxytocin studies have been recently conducted in patients with established psychosis (mostly schizophrenia)^44^. However, the results have been mixed and mostly inconclusive.

In contrast with schizophrenia, the number of intranasal oxytocin studies conducted with people at CHR-P until now is surprisingly scarce. We have previously shown that a single acute dose of intranasal oxytocin (40 IU) modulates hippocampal perfusion (a key element of our current models of the neurobiological mechanisms underlying the onset of psychosis)^45–47^ and increases the levels of choline in the anterior cingulate cortex of men at CHR-P^48^. However, while oxytocin has long been recognised for its roles in the regulation of the cardiovascular and ANSs^49–51^, we are still unclear about whether intranasal oxytocin might address the ANS dysfunction observed along the psychosis spectrum.

Oxytocin receptors are widely distributed throughout the central and peripheral ANS^52^ and in the heart^53^. Furthermore, pharmacological studies in rodents and humans have shown that oxytocin reduces blood pressure^54,55^, decreases HR^56,57^, modulates breathing^58^ and increases HRV via parasympathetic activity^59,60^. The evidence for the effects of oxytocin on HR and HRV in humans is mixed. Studies diverge between no impact of intranasal oxytocin on HR^60–63^ and decreased HR in pregnant women during oxytocin infusions^64^. Studies on HRV have reported intranasal oxytocin-induced increases at rest in healthy subjects^59,60^, patients with obstructive sleep apnoea^65^, in pregnant women after oxytocin infusion^64^, decreases during exposure to stress^66,67^ or no effects at rest in both healthy individuals^63,66^ and in men with Fragile X syndrome^68^. We have failed to detect any significant effects of a single dose of intranasal (40 IU) or intravenous infusion (10 IU) of oxytocin on HR or HRV in healthy men over an extended period of observation post dosing (14–104 min)^63^. Apart from the lack of consensus regarding the effects of oxytocin on HR/HRV in healthy humans^51^, we also lack an in-depth understanding of the effects of oxytocin on ANS cardiac regulation in clinical populations where ANS dysregulation is present^69^, such as in CHR-P.

The current proof-of-concept study comes to address this gap. We combined a single acute administration of intranasal oxytocin (40 IU) with plethysmography to investigate the effects of intranasal oxytocin on HR and HRV in CHR-P and healthy men. Focusing on people at CHR-P, who are typically antipsychotic naive^70,71^, provides an invaluable opportunity to assess HR and HRV and investigate potential intranasal oxytocin effects in a population that have a high risk of developing psychosis (in particular the brief and limited intermittent psychosis subgroup^72–74^), without the confounding effects of antipsychotic medication. We hypothesised that, compared with healthy men, CHR-P men would show increased resting HR and decreased HRV (under placebo). We further hypothesised that intranasal oxytocin (compared with placebo) would decrease HR and increase HRV in CHR-P men to levels similar to those observed in healthy men.

## Methods

### Participants

#### CHR-P sample

We recruited 30, help-seeking CHR-P men aged 18–35 from the OASIS^75^ and Tower Hamlets Early Detection Services^3^. One subject was removed due to protocol violation. We determined CHR-P status using the Comprehensive Assessment of At-Risk Mental States (CAARMS) 12/2006 criteria^76^. For further details on our exclusion criteria, see ref. ^47^. The study received National Research Ethics Service approval (14/LO/1692) and all subjects gave written informed consent.

#### Healthy comparison sample

We included data from a comparison group of 17 healthy males, aged 19–34, acquired in the context of another study. For further details on our inclusion and exclusion criteria, see ref. ^63^. Participants gave written informed consent. King’s College London Research Ethics Committee (PNM/13/14-163) approved the study.

### Study design and procedures

#### CHR-P sample

We used a randomised, double-blind, crossover single-dose challenge of intranasal oxytocin versus placebo design (1-week wash out). Participants self-administered 40 IU of intranasal oxytocin using a standard nasal spray. Our protocol followed the current practice in intranasal oxytocin pharmacological studies regarding the use of sprays to administer oxytocin^77^. It included the self-administration of one puff (4 IU) of intranasal oxytocin (Syntocinon, 40 IU/ml, Novartis, Basel, Switzerland) or matched placebo (same excipients except oxytocin) every 30 s, alternating between nostrils, until 10 puffs were administered (40 IU), during a period of 5 min. Participants were randomly allocated to a treatment order (oxytocin/placebo or placebo/oxytocin). After drug administration, participants were guided to a magnetic resonance imaging (MRI) scanner (data already reported^47,78^ or to be reported in forthcoming publications). During the MRI session, we acquired, in the following order, two arterial spin-labelling resting-state scans, two runs of BOLD fMRI during a theory-of-mind task^79^, then two structural scans (T1 and FLAIR), a resting-state BOLD fMRI and a magnetic resonance spectroscopy scan at the end. Here we report pulse plethysmography and respiratory movement data that were collected during a resting-state BOLD–fMRI scan over a period of 8 min at 62.21 ± 3.46 min post dosing.

#### Healthy comparison sample

We include data from the two arms of a randomised, double-blind, crossover single-dose challenge study^63^, where participants received 40 IU of intranasal oxytocin or placebo (same treatments and protocol as described for the CHR-P sample). After drug administration, participants were guided to an MRI scanner where we acquired a series of resting-state arterial spin labelling or BOLD–fMRI scans (data already reported^63^ or to be reported in forthcoming publications). Here we report plethysmography/respiratory data acquired during a resting-state BOLD–fMRI scan over a period of 8 min matching the post-dosing temporal window as in the CHR-P group (57.01 ± 3.38 min post dosing).

In our previous in-depth characterisation of the pharmacodynamics of intranasal oxytocin in healthy men, we have demonstrated that 40 IU intranasal oxytocin induces sustained changes in brain’s physiology and elevations in plasma oxytocin for an extended period of time post dosing^63,80^, which includes the post-dosing interval during which we sampled HR/HRV in the current study. In both datasets, subjects were asked to abstain from using recreational drugs for at least 1 week and alcohol for at least 24 h prior to each session. Breath and urine screening were conducted before each session.

### Physiological data acquisition and processing

Pulse plethysmography was continuously monitored during the resting BOLD–fMRI scan using MRI-compatible finger pulse oximetry while the participant rested in supine position, breathing spontaneously and fixating at a cross at the centre of a white screen. The data were recorded digitally as physiologic waveforms at a sampling rate of 50 Hz. Pulse plethysmography offers an easy and accurate approximation of inter-beat intervals (IBIs)^81^. When sampling rates > 25 Hz are used, time- and frequency-domain parameters of HR and HRV as assessed by pulse plethysmography are as reliable as those derived from the analysis of electrocardiogram data acquired with higher sampling rates^82^. Heart beats were firstly automatically detected using an in-house script and then visually inspected and manually cleaned for misidentified beats. IBI values were then calculated. The resulting cleaned data were then transferred to *Kubios HRV analysis software* (MATLAB, version 2 beta, Kuopio, Finland). In addition to the manual cleaning of the data, the IBI time series were processed using automatic artefact detection and detrending (using smoothing priors, *λ* = 500), and cubic spline interpolation to replace automatically detected artefacts, as provided by *Kubios*. If more than 5% of the beats required correction, we decided to exclude these datasets^83^. Then, we estimated HR and the high-frequency spectral power (0.15–0.40 Hz) of HRV (HF-HRV), using the standard *Kubios* pipeline. A detailed description of the analysis methods used to calculate these measures in *Kubios* has been provided in detail elsewhere^60,64^. We focused on the high-frequency band because this component almost exclusively reflects parasympathetic modulation of the heart rhythm^24^.

While we acquired 8 min of data in total, HR and HRV were calculated based on segments of 5 min free of artefacts (as required for pulse plethysmography to accurately reflect HRV when assessed by electrocardiography^84^). Since the data were acquired in the context of a MRI scan, we excluded the first 2 min of acquisition to account for habituation to the imaging procedure and the last minute to avoid contamination from finger movement artefacts. This choice allowed us to maximise the amount of high-quality data included in the analysis.

A chest strain gauge was used to measure respiratory movements. Strain gauge signals were manually checked for artefacts and low-pass filtered with a fourth-order, Butterworth zero-phase filter of 0.20 Hz. Then we used a technique involving cross-correlation of the filtered respiratory signal with sinusoidal signals of different frequencies to estimate the time-varying frequency of the respiration (details described elsewhere^85^).

### Statistical analysis

All statistical analyses were conducted using SPSS-25 (http://www-01.ibm.com/software/uk/analytics/spss/) and JASP (version 0.8.5.1) for the Bayesian analyses (for some primers on Bayesian inference and how to implement it with JASP, see refs. ^86–91^). Data were first examined for normality of the distributions and for the presence of outliers. When variables were not normally distributed, we applied logarithmic transformation. To test our first hypothesis, we took HR and HF-HRV from the placebo sessions and compared the CHR-P and healthy men groups using an independent sample *t* test. Since our healthy men sample was smaller, and therefore a negative finding could simply reflect the lack of sensitivity, we followed up this analysis with a Bayesian independent sample *t* test to quantify relative evidence for both the null and alternative hypotheses. We then proceeded to test our second hypothesis where we examined condition, treatment and condition × treatment effects on HR and HF-HRV in two separate linear mixed models, with a random intercept for subject and treatment and condition as fixed effects. When a significant interaction was found, we followed up with post hoc tests for simple effects, applying *Sidak* correction for multiple comparisons.

#### Post hoc analyses

We conducted a series of post hoc analyses to investigate the potential confounding effect of respiratory frequency, body mass index (BMI), age or current medication in our findings. Furthermore, we also investigated whether HR or HR-HVR relates to clinical symptomatology in CHR-P men under placebo, or whether clinical symptomatology can predict intranasal oxytocin-induced increases on HF-HRV in CHR-P men. These analyses are fully described in Supplementary Material.

In all of our analyses, we set statistical significance at *p* < 0.05 (two-tailed). For all of our Bayesian analyses, we used Cauchy (Independent sample *t* test)/beta (correlations) priors’ distributions centred around zero, with a width parameter of 1. However, we also performed robustness checks to assess sensitivity to the priors by assigning wide prior width (the plots illustrating these robustness checks can be found in Figs. S1–S3). An increase in Bayes factor (BF) in our analyses corresponds to an increase in evidence in favour of the null hypothesis. To interpret BF, we used the Lee and Wagenmakers’ classification scheme^92^: BF < 1/10, strong evidence for alternative hypothesis; 1/10 < BF < 1/3, moderate evidence for alternative hypothesis; 1/3 < BF < 1, anecdotal evidence for alternative hypothesis; BF > 1, anecdotal evidence for the null hypothesis; 3 < BF < 10, moderate evidence for the null hypothesis; BF > 10, strong evidence for the null hypothesis.

## Results

### Sample characteristics

For a brief summary of the sociodemographics of the two samples see Table 1. The two groups did not differ in age, height, weight or BMI (all *p* > 0.347). Regarding CHR-P subgroup composition (using CAARMS)^93^, our final sample included 6 men with brief limited intermittent psychotic symptoms, 22 with attenuated positive symptoms (APS) and 1 with genetic risk and deterioration. In the CHR-P group, eight men were under current pharmacological treatment (three on sertraline, one on fluoxetine, one on amitriptyline, one on mirtazapine and diazepam and one on an unknown antidepressant class). These treatments are typically employed in this group, given their high load of comorbid affective disorders^94^. Transition to psychosis was identified for 4 men within a follow-up of 26 months, but the follow-up is still ongoing. The mean CAARMS-APSs score for the CHR-P group was 11.86 (±3.35 standard deviation). Participants in either cohort could not discriminate if they had received intranasal oxytocin or placebo.

**Table 1:** Sociodemographic information (summary descriptive statistics).

		Descriptive		
Variable	Group	Mean	Standard deviation	*N* (valid)
Age	CHR-P	22.93	4.77	29
	HC	24.24	5.33	17
Height	CHR-P	178.59	8.61	27
	HC	178.15	5.59	17
Weight	CHR-P	72.35	10.62	28
	HC	74.50	8.94	17
BMI	CHR-P	22.68	3.00	27
	HC	23.41	1.97	17

In this table, we provide a statistical summary of some sociodemographic characteristics collected in both datasets. *CHR-P* clinical high-risk for psychosis, *HC* healthy men.

#### Condition, treatment, and condition × treatment effects on respiratory frequency

We did not find any significant effect of condition (*F*(1,61.84) = 3.53, *p* = 0.07), treatment (*F*(1,61.84) = 6.00 × 10^−3^, *p* = 0.94) or condition × treatment (*F*(1,61.84) = 0.05, *p* = 0.83) on respiratory frequency. In Table S1, we present the exploratory correlations between RF, HR and HF-HRV. Notably, we found a significant negative correlation between RF and HF-HRV only for CHR-P men in the oxytocin session.

### CHR-P and healthy men did not differ in HR or HF-HRV under placebo

We did not find any significant differences between healthy and CHR-P men for baseline HR (*T*(40) = −0.65, *p* = 0.52, BF = 3.47) and HF-HRV (*T*(40) = 0.68, *p* = 0.50, BF = 3.42) (Table 2).

**Table 2:** Heart rate (HR), high-frequency heart-rate variability (HF-HRV) and respiratory frequency (RF) summary descriptive statistics.

			Descriptive		
METRIC	Group	Treatment	Mean	Standard deviation	*N* (valid)
HR	CHR-P	OT	61.79	9.16	28
	CHR-P	PL	61.32	8.15	28
	HC	OT	58.81	8.19	16
	HC	PL	59.71	6.02	14
HF-HRV	CHR-P	OT	7.51	1.08	28
	CHR-P	PL	7.16	1.00	28
	HC	OT	7.19	0.96	16
	HC	PL	7.37	0.80	14
RF	CHR-P	OT	16.48	2.28	27
	CHR-P	PL	16.32	2.83	26
	HC	OT	15.36	1.85	16
	HC	PL	15.44	2.35	14

*CHR-P* clinical high-risk for psychosis, *HC* healthy men, *OT* oxytocin, *PL* placebo.

### Intranasal oxytocin (compared with placebo) increased HF-HRV in CHR-P but not in healthy men

#### Heart rate

**Fig. 1:**
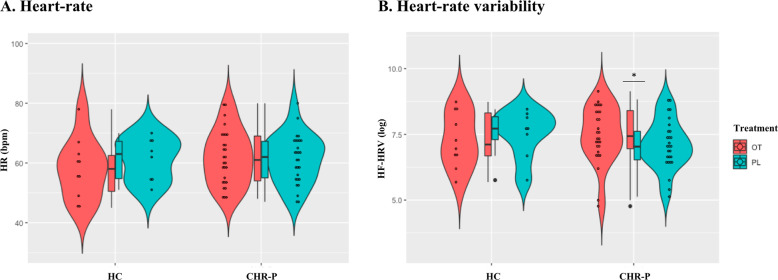
Condition, treatment, and condition × treatment effects on heart rate (a) and high-frequency heart-rate variability (b). Heart rate (HR) (**a**) and high-frequency heart-rate variability (HF-HRV) (**b**) descriptive for each treatment level within each group. Violin plots represent the smoothed distribution of the data. Box plots represent the distribution of the HR/HF-HRV values for each treatment level in each condition (median is indicated by the black central line; upper and lower whiskers denote the highest and the lowest datum within 1.5 interquartile range of the upper and lower quartiles). We tested for the main effects of condition, treatment, and the condition × treatment interaction on HR and HF-HRV using two separate linear mixed models. Statistical significance was set to *p < 0.05. Abbreviations: HC healthy controls, CHR-P clinical high-risk for psychosis, OT intranasal oxytocin, PL placebo.

We did not observe treatment (*F*(1,40.45) = 0.07, *p* = 0.79), condition (*F*(1,44.03) = 0.51, *p* = 0.48) or treatment × condition (*F*(1,40.45) = 0.33, *p* = 0.57) effects on HR (Fig. 1a).

#### High-frequency HR variability

We found a significant treatment × condition interaction on HF-HRV (*F*(1,39.93) = 7.17, *p* = 0.01). The interaction was driven by an increase in HF-HRV after intranasal oxytocin (compared with placebo) in CHR-P men (*F*(1,38.81) = 14.11, *p* = 1.00 × 10^−3^) but not in healthy men (*F*(1,40.45) = 0.40, *p* = 0.53). There were no significant differences in HF-HRV between CHR-P and healthy men under oxytocin or placebo (all *p* > 0.34). There were no significant main effects of condition (*F*(1,44.53) = 0.06, *p* = 0.81) or treatment (*F*(1,39.93) = 2.71, *p* = 0.11) on HF-HRV (Fig. 1b).

### Post hoc analyses

#### Age, BMI, respiratory frequency and current medication

Accounting for age, BMI, respiratory frequency and current medication did not affect our reported results.

#### Association between HR/HF-HRV under placebo and clinical symptomatology in CHR-P men

**Fig. 2:**
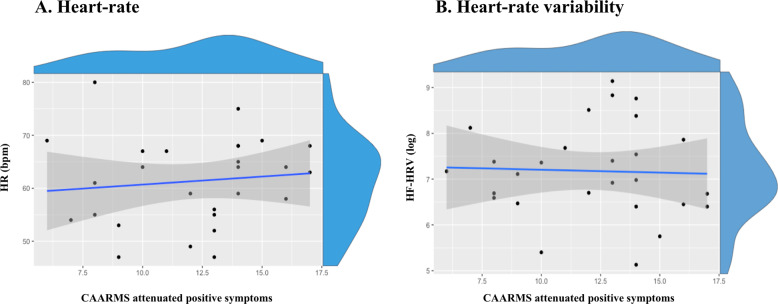
Association between heart rate (a), high-frequency heart-rate variability (b), and attenuated positive symptoms in men at clinical high risk for psychosis. Scatter plots showing the absence of correlation between heart rate (**a**) or high-frequency heart-rate variability (**b**) in the placebo session and attenuated positive symptoms (as assessed by the Comprehensive Assessment of At-risk Mental States (CAARMS)) in men at clinical high risk for psychosis. The blue line represents the fitting of a linear regression and the shadow the respective 95% confidence interval. The histograms on the top of each axis show the density distribution of each variable.

We did not find any significant correlation between HR (*r* = 0.08, *p* = 0.71, BF = 4.11) (Fig. 2a) or HF-HRV (*r* = −0.04, *p* = 0.89, BF = 3.59) (Fig. 2b) under placebo and CAARMS scores.

#### Association between clinical symptomatology and intranasal oxytocin-induced changes in HF-HRV in CHR-P men

**Fig. 3:**
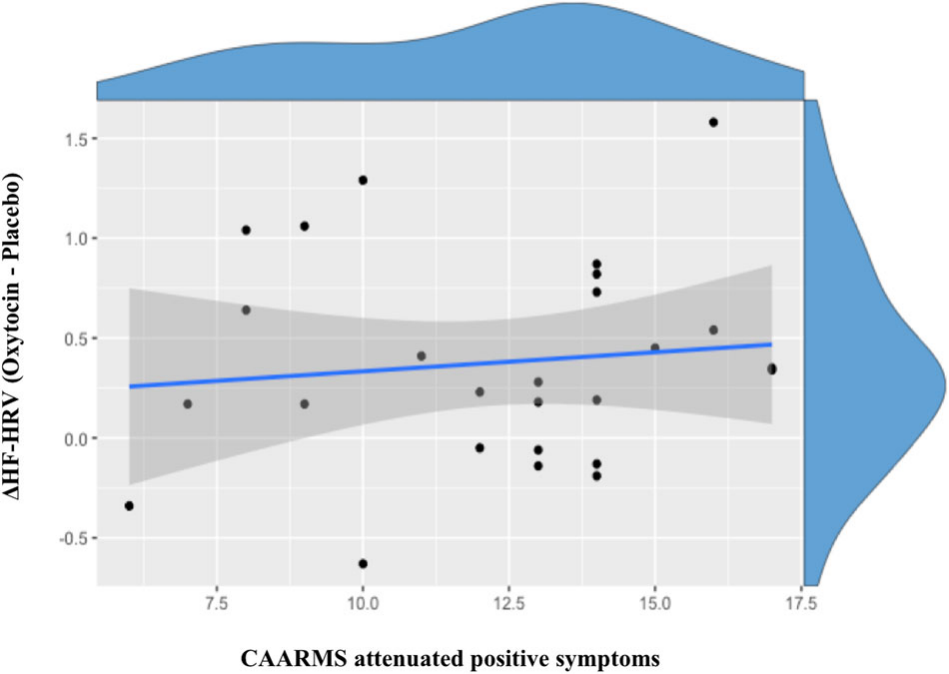
Association between high-frequency heart-rate variability response to intranasal oxytocin and attenuated positive symptoms in clinical high risk for psychosis men. Scatterplot showing the absence of correlation between the response of the high-frequency heart-rate variability (HF-HRV) to intranasal oxytocin and attenuated positive symptoms (as assessed by the Comprehensive Assessment of At-risk Mental States (CAARMS)) in men at clinical high risk for psychosis. ΔHF-HRV corresponds to the difference between the HF-HRV values of the oxytocin and placebo sessions (oxytocin–placebo). The blue line represents the fitting of a linear regression and the shadow the respective 95% confidence interval. The histograms on the top of each axis show the density distribution of each variable.

Baseline CAARMS scores did not predict intranasal oxytocin-induced changes in HF-HRV in CHR-P men (*r* = 0.11, *p* = 0.58, BF = 3.56) (Fig. 3).

## Discussion

In this study, we show that a single dose of intranasal oxytocin (compared with placebo) increases HR variability in CHR-P but not in healthy men. Nevertheless, we failed to replicate previous evidence suggesting that CHR-P, compared with healthy men, shows increased resting HR and decreased HRV. Our proof-of-concept findings support the idea that intranasal oxytocin may be of potential clinical value in improving autonomic regulation, by enhancing parasympathetic activity, during CHR-P. Given the lack of evidence for specific preventive treatments in this population, similar proof-of-concept studies that demonstrate disease-engagement targets are essential to inform future drug discovery efforts.

### Do men at clinical high risk for psychosis present alterations in HR and HRV at rest?

We did not find any significant group differences in HR or HRV between CHR-P and healthy men. The absence of significant group differences on HR and HRV was also supported by our Bayesian analysis, which showed that our null hypothesis of no differences between CHR-P and healthy men is about three times more likely than the alternative hypothesis of significant group differences (moderate evidence in favour of the null hypothesis). Our findings remained unchanged when we accounted for age, BMI, respiratory frequency or current medication status. Altogether, our findings challenge the notion that cardiac autonomic regulation is already impaired during the high-risk stages that precede the onset of full-blown psychosis, and would argue that autonomic alterations are linked to diagnosable clinical psychosis rather than constituting a general vulnerability characteristic. Consistent with this idea, in our exploratory analyses, we also did not find a correlation between psychopathology (CAARMS scores for APSs) and HR or HRV in CHR-P men.

In contrast to studies on established psychosis^32,33^, the number of studies investigating HR and HRV in CHR-P samples has been sparse. We are aware of only four studies. One study reported increased HR and decreased HRV during CHR-P and established psychosis, when compared with healthy controls and siblings, both at rest and during exposure to social stressors. The CHR-P and established psychosis groups did not differ in HR or HRV^95^. Another study found increased baseline-resting HR in CHR-P compared with low-risk controls, but did not find any difference in HRV^33^. Yet, two further studies reported no differences on HR or HRV between CHR-P and healthy controls, relatives or psychiatric controls matched for depression/anxiety, even though they reported increased HR and decreased HRV for clinically psychotic patients, compared with healthy controls^96,97^.

There are certain differences among the studies looking at HR/HRV in CHR-P (including our own study) that may help explain the inconsistent findings among them. First, there are gender differences in resting HRV (women, compared with men, present lower resting HRV^98,99^). The inclusion of samples of varied gender composition (ours focused on men) makes direct comparisons between studies challenging, especially if there is a gender-by-treatment interaction regarding the effects of HR/HRV. Second, in the two studies^33,95^ where differences were evident at rest, participants had been exposed to social stressor paradigms that might have elicited anticipatory stress responses during the “resting” HR/HRV measurements. These anticipatory responses may have amplified the differences between the CHR-P/established psychosis and the healthy controls/siblings’ groups. In our study, the plethysmography data were acquired at rest—but participants were in the MRI environment, which could have been perceived as distressing^100^. To account for habituation to the imaging procedure, we excluded the first 2 min of recording. Therefore, we believe that our experimental setup resembles better the one used in the latter two studies^96,97^ that also reported no resting HR/HRV differences between CHR-P and healthy individuals/relatives. Third, in our study, there was a difference in the procedure for data acquisition between the CHR-P and healthy groups that should be considered. In the healthy group, participants had been cannulated for repeated blood sampling, and a small blood sample (5 ml) had been drawn preceding the resting BOLD–fMRI scan. This procedure was not present in our CHR-P protocol. It is possible that this procedure added discomfort/perceived distress or compensatory autonomic responses to healthy participants that could have attenuated existent HR/HRV differences from CHR-P men. Finally, in our study, we measured HR/HRV, in both groups, after a long scanning period during which participants laid in supine position. In studies where HR/HRV have been reported to differ between CHR-P and healthy controls, recordings were done in the sitting/orthostatic position^33,95^. Body position has been shown to affect cardiac autonomic regulation^101^. Therefore, we cannot also exclude the contribution of this factor in mitigating potential existent differences between groups on HR/HRV, if they existed.

### Can intranasal oxytocin increase resting HRV in CHR-P men?

We did not detect a treatment or treatment × condition effect on HR. However, we found a significant treatment × condition effect on HF-HRV, reflecting an increase in HF-HRV after intranasal oxytocin (compared with placebo) in CHR-P but not in healthy men. Importantly, our findings remained unaltered after accounting for age, BMI, current medication or respiratory frequency. Intranasal oxytocin-induced increases in HF-HRV were not predicted by the severity of APSs. Altogether, our findings suggest that intranasal oxytocin (40 IU), compared with placebo, increases cardio-parasympathetic activity in CHR-P men, irrespective of the severity of the APSs.

The absence of the effects of intranasal oxytocin on HR in the current study is not fully surprising. Apart from one study, which was conducted in pregnant women during continuous infusion of oxytocin, that reported bradycardic effects^102^, four other studies (including one of our research group using the same healthy cohort^63^) have shown that intranasal oxytocin (40 IU) (compared with placebo) does not affect HR in humans^60–62^. In animal models, the evidence for the effects of oxytocin on HR diverges between decreases^103^, increases^58^ and no effects^104^. A lack of change in HR after oxytocin administration has been interpreted in the context of putative stimulatory effects on both branches of the ANS, which may cancel each other out^62,104^. Since we did not assess cardiac sympathetic regulation in our sample, we cannot say whether the same applies to our findings. Furthermore, we also should acknowledge that it is difficult to interpret HR data (in our study and in others) without considering blood pressure, as changes in HR may be compensatory^105^. This aspect should be taken into consideration in future studies revisiting this question.

The evidence for the effects of oxytocin on HRV in humans has been mixed. Studies on HRV have reported intranasal oxytocin-induced increases at rest in healthy subjects^59,60^ and patients with obstructive sleep apnoea^65^, and in pregnant women after oxytocin infusion^64^, decreases during exposure to stress^66,67^ or no effects at rest in both healthy individuals^63,66^ and in men with Fragile X syndrome^68^. Our own work has failed to detect any significant effects of a single dose of intranasal (40 IU) or intravenous (10 IU) oxytocin on resting HRV in healthy men over an extended period of observation post dosing^63^.

There are some plausible hypotheses that might explain apparent discrepancies in the literature regarding the effects of intranasal oxytocin on HR or HRV in healthy participants, or differences between clinical and healthy groups in the oxytocin treatment response. We discuss three of these hypotheses, noting that some predictions may be contradictory and hence require further research. First, following current models of the pharmacodynamics of intranasal oxytocin in humans, which have suggested an inverted U-shape curve of response^106,107^, it is tempting to speculate that our dose (40 IU) may have been higher than the optimal dose to achieve increases in HRV in healthy men. Indeed, the two studies reporting significant increases after intranasal oxytocin in healthy men used doses of 20–24 IU. We need studies to examine the dose–response effects of intranasal oxytocin on HRV in humans. Second, the oxytocin signalling pathway may be more sensitive to exogenous oxytocin in CHR-P men. This hypothesis is consistent with evidence from two studies. The first study reported increased oxytocin and oxytocin receptor mRNA expression in peripheral blood lymphocytes in first-episode schizophrenia patients when compared with healthy controls^108^. The second study reported decreased methylation of the oxytocin receptor gene promoter, which would typically result in increased oxytocin receptor expression, in peripheral blood lymphocytes of women with a recent schizophrenia onset or at CHR-P, when compared with healthy women^109^. In contrast, there is also evidence of region-specific decreases in oxytocin receptor expression (in the temporal cortex/cerebellum) in the brain of patients with established psychosis, compared with healthy controls^110^. To the extent that such alterations are present in CHR-P in systems involved in the regulation of HR/HRV, this might predict an attenuated HR/HRV response to exogenous oxytocin and hence, if an inverted U-shape dose–response model is true, implying that an increased dose (compared with healthy men) is required in CHR-P men to achieve an optimal effect. While plausible, these hypotheses remain speculatory at the moment and therefore require further research.

Currently, it is unclear if the effects of oxytocin on HRV should be attributed to direct actions on peripheral elements of the ANS and cardiovascular systems, whether they are mediated via actions on central targets that regulate peripheral ANS activity, such as the amygdala (a brain area often implicated in the effects of intranasal oxytocin^111^), or both. Future studies combining the concomitant administration of oxytocin and a non-brain receptor antagonist with neuroimaging and physiological recordings may help us to disambiguate this question.

### Limitations

First, we note that while our primary hypotheses were a priori (i.e., they were specified before conducting any analyses, and we are explicit about the instances when they are not), they were not part of the initial study protocols. Therefore, power calculations for these specific analyses had not been conducted a priori. Recognising the importance that the issue of statistical might have for the appraisal of our findings, we conducted some post hoc power analyses to investigate what is the lowest effect size our samples would have allowed us to detect with an acceptable statistical power of 80% in two-tailed tests for each of our main hypothesis (which we present in Fig. S4). For our first hypothesis (differences in HR and HRV between healthy and men at CHR-P under placebo), we estimated that our samples (HC: *n* = 14 valid cases; CHR-P: *n* = 28 valid cases) would have allowed us to detect, using an independent sample *t* test, a large effect size of *d* = 0.94 (Fig. S4A). Given that differences between healthy and CHR-P individuals on HR or HRV have been previously reported within the *d* = 0.20–0.50 range^112^, our study was underpowered to test our first hypothesis. We used Bayesian statistics to quantify the relative evidence favouring the null and alternative hypotheses to explore this question further. We found that our data support the null hypothesis of no differences between CHR-P and healthy men on HR/HRV under placebo (even though the evidence in favour of the null hypothesis was only moderate). For our second hypothesis (effects of intranasal oxytocin on HR and HRV in men at CHR-P), our sample size (intranasal oxytocin: *n* = 28 valid cases; placebo: *n* = 28 valid cases) would have allowed us to detect, using a two-sided paired *t* test, a medium effect size of *d* = 0.55 (Fig. S4B). Given that a previous study has reported effect sizes for intranasal oxytocin-induced increases in HRV within the *d* = 0.20–0.50 range^113^, our sample size for testing the second hypothesis was reasonable. Given the power constraints of this study, our findings should be considered preliminary and we encourage replication studies.

Second, we only included men, which limits our ability to extrapolate our findings to women—especially when the effects of intranasal oxytocin may vary across genders^114^. Third, our findings are limited to the dose (40 IU), regime of administration (single) and time post dosing we employed. Future studies should investigate a wider range of doses, different regimes of administration (single vs. chronic) and time course of these effects. Fourth, while we could exclude the potential confounding effect of respiratory frequency on our findings, we could not perform an in-depth investigation of the respiratory dynamics, including respiratory depth^115^. Therefore, we cannot exclude the potential contribution of this confound. Fifth, our physiological data were acquired during an MRI scan. It is possible that the distress associated with the MRI environment might have affected our findings somehow (e.g., by attenuating differences between CHR-P and healthy men or exacerbating the effects of intranasal oxytocin in men at CHR-P, who might perceive the MRI environment as particularly distressing). Future studies should attempt to replicate our findings outside of the MRI environment. Finally, in the CHR-P group, participants had performed a theory-of-mind task^79^, followed by two structural scans, before the beginning of the resting-state BOLD–fMRI data. Our protocol for the healthy sample only included resting-state scans. While the inclusion of the two structural scans (total duration ~7 min) between the task and the resting-state BOLD–fMRI scan is likely to have minimised any potential transference effects, we cannot exclude a potential contribution of task × intranasal oxytocin interaction to the significant increases in HRV we report herein for CHR-P men.

## Conclusion

In this proof-of-concept study, we show that intranasal oxytocin (40 IU) increases cardio-parasympathetic activity in CHR-P but not in healthy men, demonstrating a potential disease-engagement psychopharmacological target. Our findings support the need to investigate further intranasal oxytocin as an intervention to improve ANS regulation during the high-risk stage of psychosis. Heightened ANS response to psychosocial stressors has been suggested to contribute to psychosis and accompanying increased cardiovascular risk. Hence, it is conceivable that, by increasing cardio-parasympathetic activity, intranasal oxytocin might hold promise to prevent transition into full-blown psychosis and/or relapse and address potential cardiovascular comorbidities^2^ in patients with psychosis or at risk. This hypothesis should be addressed in further longitudinal clinical studies investigating the effects of chronic administrations of oxytocin. Furthermore, given that oxytocin might also improve other deficits of CHR-P, such as social functioning, it is tempting to speculate that intranasal oxytocin could constitute an innovative treatment, with multiple potential therapeutic benefits cutting across different symptom domains, and minimal side effects for people at CHR-P. In the absence of specific preventive treatments in this population, similar proof-of-concept studies are essential to inform and guide potential future therapeutic advances.

## Supplementary information

Supplementary Information

## References

[1] Oh H, Koyanagi A, Kelleher I, DeVylder J (2018). Psychotic experiences and disability: findings from the Collaborative Psychiatric Epidemiology Surveys. Schizophr. Res..

[2] Fusar-Poli P, McGorry PD, Kane JM (2017). Improving outcomes of first-episode psychosis: an overview. World Psychiatry.

[3] Fusar-Poli P (2019). Pan-London Network for psychosis-prevention (PNP). Front. Psychiatry.

[4] Salazar de Pablo, G., Catalan, A. & Fusar-Poli, P. Clinical validity of DSM-5 attenuated psychosis syndrome: advances in diagnosis, prognosis, and treatment. *JAMA Psychiatry.*10.1001/jamapsychiatry.2019.3561 (2019). 10.1001/jamapsychiatry.2019.3561 31746950

[5] Davies C (2018). Efficacy and acceptability of interventions for attenuated positive psychotic symptoms in individuals at clinical high risk of psychosis: a network meta-analysis. Front. Psychiatry.

[6] Fusar-Poli P (2017). The Clinical High-Risk State for Psychosis (CHR-P), Version II. Schizophr. Bull..

[7] Oliver D (2020). What causes the onset of psychosis in individuals at clinical high risk? A meta-analysis of risk and protective factors. Schizophr. Bull..

[8] Radua J (2018). What causes psychosis? An umbrella review of risk and protective factors. World Psychiatry.

[9] Fusar-Poli P, Raballo A, Parnas J (2017). What is an attenuated psychotic symptom? on the importance of the context. Schizophr. Bull..

[10] Fusar-Poli P (2015). Disorder, not just state of risk: meta-analysis of functioning and quality of life in people at high risk of psychosis. Br. J. Psychiatry.

[11] Falkenberg I (2015). Why are help-seeking subjects at ultra-high risk for psychosis help-seeking?. Psychiatry Res..

[12] Fusar-Poli P (2013). The psychosis high-risk state a comprehensive state-of-the-art review. JAMA Psychiatry.

[13] Nuechterlein KH, Dawson ME (1984). A heuristic vulnerability/stress model of schizophrenic episodes. Schizophr. Bull..

[14] Zubin J, Spring B (1977). Vulnerability-a new view of schizophrenia. J. Abnorm. Psychol..

[15] van Winkel R, Stefanis NC, Myin-Germeys I (2008). Psychosocial stress and psychosis. A review of the neurobiological mechanisms and the evidence for gene-stress interaction. Schizophr. Bull..

[16] Pinckaers FME (2019). Evidence for interaction between genetic liability and childhood trauma in the development of psychotic symptoms. Soc. Psychiatry Psychiatr. Epidemiol..

[17] Collip D, Myin-Germeys I, Van Os J (2008). Does the concept of “sensitization” provide a plausible mechanism for the putative link between the environment and schizophrenia?. Schizophr. Bull..

[18] Bloomfield, M. A., McCutcheon, R. A., Kempton, M., Freeman, T. P. & Howes, O. The effects of psychosocial stress on dopaminergic function and the acute stress response. *Elife*. 10.7554/eLife.46797 (2019). 10.7554/eLife.46797 PMC6850765 31711569

[19] Dahoun T (2019). The relationship between childhood trauma, dopamine release and dexamphetamine-induced positive psychotic symptoms: a [(11)C]-(+)-PHNO PET study. Transl. Psychiatry.

[20] Clamor A, Lincoln TM, Thayer JF, Koenig J (2016). Resting vagal activity in schizophrenia: meta-analysis of heart rate variability as a potential endophenotype. Br. J. Psychiatry.

[21] Bar K (2018). Prevalence and consequences of cardiac autonomic dysfunction (Cadf) in 112 unmedicated patients with schizophrenia. Schizophr. Bull..

[22] Bar KJ (2015). Cardiac autonomic dysfunction in patients with schizophrenia and their healthy relatives-a small review. Front. Neurol..

[23] Davies C (2018). Efficacy and acceptability of interventions for attenuated positive psychotic symptoms in individuals at clinical high risk of psychosis: a network meta-analysis. Front. Psychiatry.

[24] Shaffer F, Ginsberg JP (2017). An overview of heart rate variability metrics and norms. Front. Public Health.

[25] Berntson GG, Cacioppo JT, Quigley KS, Fabro VT (1994). Autonomic space and psychophysiological response. Psychophysiology.

[26] Zahn TP, Pickar D (1993). Autonomic effects of clozapine in schizophrenia: comparison with placebo and fluphenazine. Biol. Psychiatry.

[27] Bar KJ (2005). Loss of efferent vagal activity in acute schizophrenia. J. Psychiatr. Res..

[28] Schulz S (2013). Cardiovascular coupling analysis with high-resolution joint symbolic dynamics in patients suffering from acute schizophrenia. Physiol. Meas..

[29] Bar KJ (2010). Autonomic dysfunction in unaffected first-degree relatives of patients suffering from schizophrenia. Schizophr. Bull..

[30] Berger S (2010). Reduced cardio-respiratory coupling indicates suppression of vagal activity in healthy relatives of patients with schizophrenia. Prog. Neuropsychopharmacol. Biol. Psychiatry.

[31] Abhishekh HA (2014). Prolonged reaction to mental arithmetic stress in first-degree relatives of schizophrenia patients. Clin. Schizophr. Relat. Psychoses.

[32] Counotte J (2016). High psychosis liability is associated with altered autonomic balance during exposure to virtual reality social stressors. Early Inter. Psychia.

[33] Weintraub MJ (2019). Affective and physiological reactivity to emotional comments in individuals at elevated risk for psychosis. Schizophr. Res..

[34] Feifel D (2010). Adjunctive intranasal oxytocin reduces symptoms in schizophrenia patients. Biol. Psychiatry.

[35] Lee MR (2013). Effects of adjunctive intranasal oxytocin on olfactory identification and clinical symptoms in schizophrenia: results from a randomized double blind placebo controlled pilot study. Schizophr. Res..

[36] Ota M, Yoshida S, Nakata M, Yada T, Kunugi H (2018). The effects of adjunctive intranasal oxytocin in patients with schizophrenia. Postgrad. Med..

[37] MacDonald E (2011). A review of safety, side-effects and subjective reactions to intranasal oxytocin in human research. Psychoneuroendocrinology.

[38] DeMayo MM, Song YJC, Hickie IB, Guastella AJ (2017). A review of the safety, efficacy and mechanisms of delivery of nasal oxytocin in children: therapeutic potential for autism and Prader-Willi syndrome, and recommendations for future research. Paediatr. Drugs.

[39] Verhees M (2018). No side-effects of single intranasal oxytocin administration in middle childhood. Psychopharmacology.

[40] den Boer JA, Westenberg HG (1992). Oxytocin in obsessive compulsive disorder. Peptides.

[41] Epperson CN, McDougle CJ, Price LH (1996). Intranasal oxytocin in obsessive-compulsive disorder. Biol. Psychiatry.

[42] Fusar-Poli P (2019). Preventive treatments for psychosis: umbrella review (just the evidence). Front. Psychiatry.

[43] Davies C (2018). Lack of evidence to favor specific preventive interventions in psychosis: a network meta-analysis. World Psychiatry.

[44] Williams DR, Burkner PC (2017). Effects of intranasal oxytocin on symptoms of schizophrenia: A multivariate Bayesian meta-analysis. Psychoneuroendocrinology.

[45] Allen P (2018). Increased resting hippocampal and basal ganglia perfusion in people at ultra high risk for psychosis: replication in a second cohort. Schizophr. Bull..

[46] Allen P (2016). Resting hyperperfusion of the hippocampus, midbrain, and basal ganglia in people at high risk for psychosis. Am. J. Psychiatry.

[47] Davies C (2019). Oxytocin modulates hippocampal perfusion in people at clinical high risk for psychosis. Neuropsychopharmacology.

[48] Davies C (2019). Neurochemical effects of oxytocin in people at clinical high risk for psychosis. Eur. Neuropsychopharmacol..

[49] Japundzic-Zigon N (2013). Vasopressin and oxytocin in control of the cardiovascular system. Curr. Neuropharmacol..

[50] Gutkowska J, Jankowski M (2012). Oxytocin revisited: its role in cardiovascular regulation. J. Neuroendocrinol..

[51] Quintana DS, Kemp AH, Alvares GA, Guastella AJ (2013). A role for autonomic cardiac control in the effects of oxytocin on social behavior and psychiatric illness. Front. Neurosci..

[52] Landgraf R, Neumann ID (2004). Vasopressin and oxytocin release within the brain: a dynamic concept of multiple and variable modes of neuropeptide communication. Front. Neuroendocrinol..

[53] Jankowski M, Hajjar F, Mukaddam-Daher S, McCann SM, Gutkowska J (1999). Rat heart: a site of oxytocin production and action. FASEB J..

[54] Woodbury RA, Abreu BE (1944). Influence of oxytocin (pitocin) upon the heart and blood pressure of the chicken, rabbit, cat, dog and turtle. Am. J. Physiol..

[55] Woodbury RA, Hamilton WF, Volpitto PP, Abreu BE, Harper HT (1944). Cardiac and blood pressure effects of pitocin (oxytocin) in man. J. Pharmacol. Exp. Therap..

[56] Grippo AJ, Trahanas DM, Zimmerman RR, Porges SW, Carter CS (2009). Oxytocin protects against negative behavioral and autonomic consequences of long-term social isolation. Psychoneuroendocrinology.

[57] Higa KT, Mori E, Viana FF, Morris M, Michelini LC (2002). Baroreflex control of heart rate by oxytocin in the solitary-vagal complex. Am. J. Physiol..

[58] Mack SO (2002). Paraventricular oxytocin neurons are involved in neural modulation of breathing. J. Appl. Physiol..

[59] Norman GJ (2011). Oxytocin increases autonomic cardiac control: moderation by loneliness. Biol. Psychol..

[60] Kemp AH (2012). Oxytocin increases heart rate variability in humans at rest: implications for social approach-related motivation and capacity for social engagement. PLoS ONE.

[61] Hall SS, Lightbody AA, McCarthy BE, Parker KJ, Reiss AL (2012). Effects of intranasal oxytocin on social anxiety in males with fragile X syndrome. Psychoneuroendocrinology.

[62] Norman GJ (2011). Oxytocin increases autonomic cardiac control: moderation by loneliness. Biol. Psychol..

[63] Martins, D. A. et al. Effects of route of administration on oxytocin-induced changes in regional cerebral blood flow in humans. *Nat. Commun* **11**, 1160 (2020). 10.1038/s41467-020-14845-5 PMC7054359 32127545

[64] Weissman A, Tobia RS, Burke YZ, Maxymovski O, Drugan A (2017). The effects of oxytocin and atosiban on the modulation of heart rate in pregnant women. J. Matern. Fetal Neonatal Med..

[65] Jain V (2017). Benefits of oxytocin administration in obstructive sleep apnea. Am. J. Physiol. Lung Cell Mol. Physiol..

[66] Tracy LM, Gibson SJ, Labuschagne I, Georgiou-Karistianis N, Giummarra MJ (2018). Intranasal oxytocin reduces heart rate variability during a mental arithmetic task: a randomised, double-blind, placebo-controlled cross-over study. Prog. Neuropsychopharmacol. Biol. Psychiatry.

[67] Riem MME, Kunst LE, Bekker MHJ, Fallon M, Kupper N (2019). Intranasal oxytocin enhances stress-protective effects of social support in women with negative childhood experiences during a virtual Trier Social Stress Test. Psychoneuroendocrinology.

[68] Hall SS, Lightbody AA, McCarthy BE, Parker KJ, Reiss AL (2012). Effects of intranasal oxytocin on social anxiety in males with fragile X syndrome. Psychoneuroendocrinology.

[69] Reiss AB (2019). Oxytocin: Potential to mitigate cardiovascular risk. Peptides.

[70] Fusar-Poli P (2015). Antidepressant, antipsychotic and psychological interventions in subjects at high clinical risk for psychosis: OASIS 6-year naturalistic study. Psychol. Med..

[71] Fusar-Poli, P. et al. Prevention of psychosis: advances in detection, prognosis, and intervention. *JAMA Psychiatry*. 10.1001/jamapsychiatry.2019.4779 (2020). 10.1001/jamapsychiatry.2019.4779 32159746

[72] Fusar-Poli P (2016). Prognosis of brief psychotic episodes: a meta-analysis. JAMA Psychiatry.

[73] Fusar-Poli P (2017). Diagnostic and prognostic significance of brief limited intermittent psychotic symptoms (BLIPS) in individuals at ultra high risk. Schizophr. Bull..

[74] Fusar-Poli P (2019). Unmet needs for treatment in 102 individuals with brief and limited intermittent psychotic symptoms (BLIPS): implications for current clinical recommendations. Epidemiol. Psychiatr. Sci..

[75] Fusar-Poli P, Byrne M, Badger S, Valmaggia LR, McGuire PK (2013). Outreach and support in south London (OASIS), 2001-2011: ten years of early diagnosis and treatment for young individuals at high clinical risk for psychosis. Eur. Psychiatry.

[76] Yung AR, Yuen HP, Phillips LJ, Francey S, McGorry PD (2003). Mapping the onset of psychosis: the comprehensive assessment of at risk mental states (CAARMS). Schizophr. Res..

[77] Guastella AJ (2013). Recommendations for the standardisation of oxytocin nasal administration and guidelines for its reporting in human research. Psychoneuroendocrinology.

[78] Davies C (2019). Neurochemical effects of oxytocin in people at clinical high risk for psychosis. Eur. Neuropsychopharmacol..

[79] Aoki Y (2014). Oxytocin improves behavioural and neural deficits in inferring others’ social emotions in autism. Brain.

[80] Paloyelis Y (2016). A spatiotemporal profile of in vivo cerebral blood flow changes following intranasal oxytocin in humans. Biol. Psychiatry.

[81] Schafer A, Vagedes J (2013). How accurate is pulse rate variability as an estimate of heart rate variability? A review on studies comparing photoplethysmographic technology with an electrocardiogram. Int. J. Cardiol..

[82] Choi A, Shin H (2017). Photoplethysmography sampling frequency: pilot assessment of how low can we go to analyze pulse rate variability with reliability?. Physiol. Meas..

[83] Billman GE, Huikuri HV, Sacha J, Trimmel K (2015). An introduction to heart rate variability: methodological considerations and clinical applications. Front. Physiol..

[84] Selvaraj N, Jaryal A, Santhosh J, Deepak KK, Anand S (2008). Assessment of heart rate variability derived from finger-tip photoplethysmography as compared to electrocardiography. J. Med. Eng. Technol..

[85] Chuen L, Sears D, Mcadams S (2016). Psychophysiological responses to auditory change. Psychophysiology.

[86] Rouder JN, Haaf JM, Vandekerckhove J (2018). Bayesian inference for psychology, part IV: parameter estimation and Bayes factors. Psychon. Bull. Rev..

[87] Matzke D, Boehm U, Vandekerckhove J (2018). Bayesian inference for psychology, part III: parameter estimation in nonstandard models. Psychon. Bull. Rev..

[88] Wagenmakers EJ (2018). Bayesian inference for psychology. Part I: theoretical advantages and practical ramifications. Psychon. Bull. Rev..

[89] Wagenmakers EJ (2018). Bayesian inference for psychology. Part II: example applications with JASP. Psychon. Bull. Rev..

[90] Etz A, Vandekerckhove J (2018). Introduction to Bayesian inference for psychology. Psychon. Bull. Rev..

[91] Quintana DS, Williams DR (2018). Bayesian alternatives for common null-hypothesis significance tests in psychiatry: a non-technical guide using JASP. BMC Psychiatry.

[92] Lee MD, W. E.-J. *Bayesian Cognitive Modeling: A Practical Course*. (Cambridge: Cambridge University Press, 2014).

[93] Fusar-Poli P (2016). Heterogeneity of psychosis risk within individuals at clinical high risk: a meta-analytical stratification. JAMA Psychiatry.

[94] Fusar-Poli P, Nelson B, Valmaggia L, Yung AR, McGuire PK (2014). Comorbid depressive and anxiety disorders in 509 individuals with an at-risk mental state: impact on psychopathology and transition to psychosis. Schizophr. Bull..

[95] Counotte J (2017). High psychosis liability is associated with altered autonomic balance during exposure to Virtual Reality social stressors. Schizophr. Res..

[96] Clamor A (2014). Altered autonomic arousal in psychosis: an analysis of vulnerability and specificity. Schizophr. Res..

[97] Clamor A, Sundag J, Lincoln TM (2019). Specificity of resting-state heart rate variability in psychosis: a comparison with clinical high risk, anxiety, and healthy controls. Schizophr. Res..

[98] Huang WL (2013). Gender differences in personality and heart-rate variability. Psychiatry Res..

[99] Saleem S, Hussain MM, Majeed SM, Khan MA (2012). Gender differences of heart rate variability in healthy volunteers. J. Pak. Med. Assoc..

[100] Flaherty JA, Hoskinson K (1989). Emotional distress during magnetic-resonance imaging. N. Engl. J. Med..

[101] Watanabe N, Reece J, Polus BI (2007). Effects of body position on autonomic regulation of cardiovascular function in young, healthy adults. Chiropr. Osteopat..

[102] Weissman A, Tobia RS, Burke YZ, Maxymovski O, Drugan A (2017). The effects of oxytocin and atosiban on the modulation of heart rate in pregnant women. J. Matern. Fetal Neonatal Med..

[103] Mukaddam-Daher S, Yin YL, Roy J, Gutkowska J, Cardinal R (2001). Negative inotropic and chronotropic effects of oxytocin. Hypertension.

[104] Holst S, Uvnas-Moberg K, Petersson M (2002). Postnatal oxytocin treatment and postnatal stroking of rats reduce blood pressure in adulthood. Autonomic Neurosci..

[105] Thames MD, Kontos HA (1970). Mechanisms of baroreceptor-induced changes in heart rate. Am. J. Physiol..

[106] Spengler FB (2017). Kinetics and dose dependency of intranasal oxytocin effects on amygdala reactivity. Biol. Psychiatry.

[107] Quintana DS (2016). Low dose intranasal oxytocin delivered with Breath Powered device dampens amygdala response to emotional stimuli: a peripheral effect-controlled within-subjects randomized dose-response fMRI trial. Psychoneuroendocrinology.

[108] Yang X (2017). Up-regulated expression of oxytocin mRNA in peripheral blood lymphocytes from first-episode schizophrenia patients. Oncotarget.

[109] Bang M (2019). Reduced DNA methylation of the oxytocin receptor gene is associated with anhedonia-asociality in women with recent-onset schizophrenia and ultra-high risk for psychosis. Schizophr. Bull..

[110] Uhrig S (2016). Reduced oxytocin receptor gene expression and binding sites in different brain regions in schizophrenia: a post-mortem study. Schizophr. Res..

[111] Rosenfeld AJ, Lieberman JA, Jarskog LF (2011). Oxytocin, dopamine, and the amygdala: a neurofunctional model of social cognitive deficits in schizophrenia. Schizophr. Bull..

[112] Counotte J (2017). High psychosis liability is associated with altered autonomic balance during exposure to Virtual Reality social stressors. Schizophr. Res..

[113] Kemp AH (2012). Oxytocin increases heart rate variability in humans at rest: implications for social approach-related motivation and capacity for social engagement. PLoS ONE.

[114] Bolea-Alamanac B, Bailey SJ, Lovick TA, Scheele D, Valentino R (2018). Female psychopharmacology matters! Towards a sex-specific psychopharmacology. J. Psychopharmacol..

[115] Quintana DS, Alvares GA, Heathers JAJ (2016). Guidelines for Reporting Articles on Psychiatry and Heart rate variability (GRAPH): recommendations to advance research communication. Transl. Psychiatry.

