# Supplementary material for: Intranasal oxytocin increases heart-rate variability in men at clinical high risk for psychosis: a proof-of-concept study
Source: Transl Psychiatry. 2020 Jul 12;10:227. doi: 10.1038/s41398-020-00890-7 (PMC7354990; doi:10.1038/s41398-020-00890-7)
Supplement: Supplementary file 1 — Supplementary Information [file 41398_2020_890_MOESM1_ESM.docx]

**Supplementary Material**

**Statistical analysis**

**Post-hoc analyses**

**Respiratory dynamics:** Respiratory dynamics parameters such as frequency and depth are related to heart rate dynamics. Changes in respiratory patterns can influence both HR and HRV independently of cardiac autonomic activity^1-3^. Therefore, the measurement of spontaneous respiration rate is often recommended in HRV studies^3^. Correcting for respiratory frequency has been recommended in patients with severe psychiatric illness^4^ or under pharmacological manipulations with unknown effects on cardiorespiratory dynamics. Therefore, we first examined respiratory frequencies for each participant/session to make sure they lied within the range where normal cardiorespiratory assumptions are not violated if conventional HF-HRV bands are used^3^. We also tested for condition, treatment and condition x treatment effects on respiratory frequency to rule out this potential confound. As a final sanity check, we also calculated Pearson correlations (bootstrapping, 1000 samples) and its Bayesian counterpart between RF, HR and HF-HRV, separately for each condition and treatment level – these correlations could offer some insight on whether the confounding effect of RF in our analyses is a justifiable concern.

**Age, BMI and current medication:** Given the known effects of age^5^ and BMI^6^ on heart rate variability and the fact that some of our CHR-P participants were under pharmacological treatment (which may also impact on HR and HRV^7^), we then repeated all of our analyses including body-mass index (BMI), age, respiratory frequency and current medication as covariates in our models to examine the robustness of our findings.

**Association between HR/HF-HRV under placebo and clinical symptomatology in CHR-P men:** We also performed exploratory Pearson correlations (using bootstrap, 1000 samples) between HR and HF-HRV in CHRP-men under placebo, and clinical symptomatology as assessed by the CAARMS attenuated positive symptoms scores.

**Association between clinical symptomatology and intranasal oxytocin-induced changes in HF-HRV in CHR-P men:** To investigate whether CHR-P men with higher symptomatology would benefit the most from oxytocin, we also explored whether CAARMS attenuated positive symptoms scores could predict intranasal oxytocin-induced effects on HF-HRV in CHR-P men by running Pearson correlations (using bootstrap, 1000 samples) using the difference in HF-HRV between the intranasal oxytocin and placebo sessions. We repeated both correlation analyses using Bayesian Pearson correlation.

**Results**

**Fig. S1 – Bayesian comparison of heart rate (A) and high-frequency heart rate variability (B) between healthy men and men at clinical high-risk for psychosis during the placebo session (priors).** In this figure, we show the results of a robustness check illustrating the effects of assigning a range of stretched prior widths on Bayes factor values for the comparison of heart rate (A) and high-frequency heart rate variability (B) between healthy and men at clinical high-risk for psychosis during the placebo session.


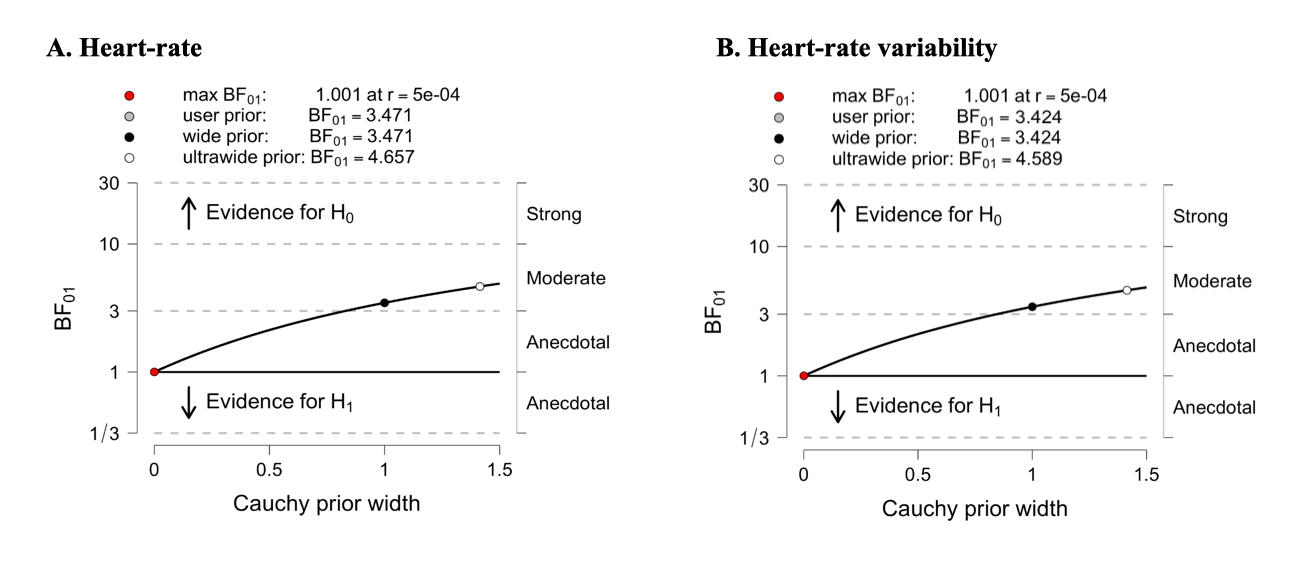


**Fig. S2 – Bayesian correlations between heart rate (A), high-frequency heart rate variability (B) and attenuated positive symptoms in men at clinical high-risk for psychosis during the placebo session (priors).** In this figure, we show the results of a robustness check illustrating the effects of assigning a range of stretched prior widths on Bayes factor values for the correlations between heart rate (A), high-frequency heart rate variability (B) and attenuated positive symptoms in men at clinical high-risk for psychosis during the placebo session.


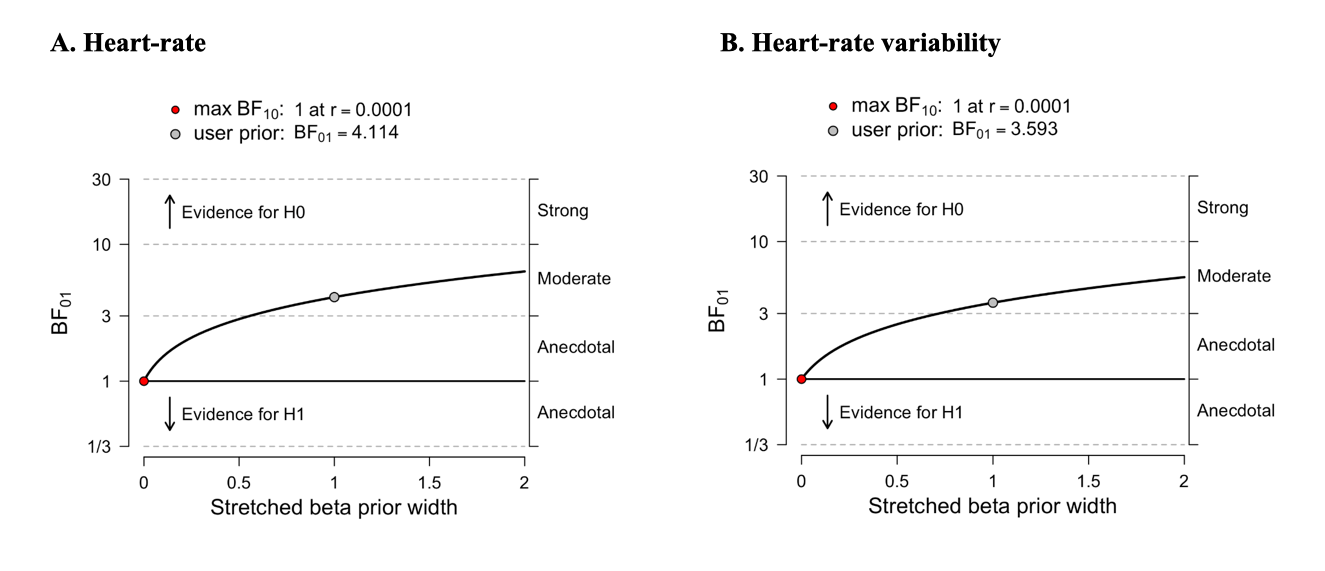


**Fig. S3 – Bayesian correlations between high-frequency heart rate variability response to intranasal oxytocin and attenuated positive symptoms in men at clinical high-risk for psychosis men (priors).** In this figure, we show the results of a robustness check illustrating the effects of assigning a range of stretched prior widths on Bayes factor values for the correlations between the response of high-frequency heart rate variability to intranasal oxytocin and attenuated positive symptoms in men at clinical high-risk for psychosis.


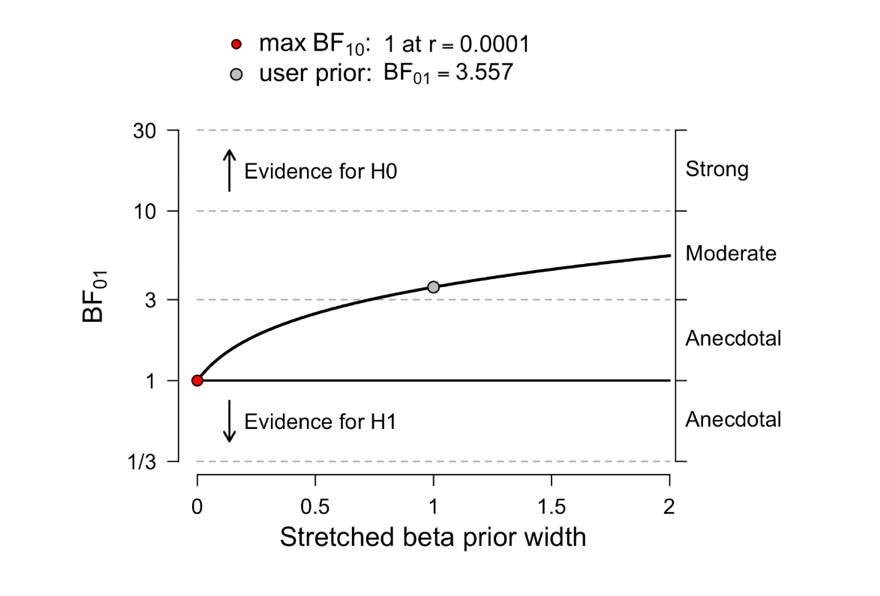


**Table S1. Association between respiratory frequency and heart rate or high frequency heart rate variability.** We show the results of Pearson correlations (bootstrapping 1000 samples) and its Bayesian counterpart to investigate associations between respiratory frequency (RF) and heart rate (HR) or high frequency heart rate variability (HF-HRV) for each group and treatment levels. R refers to Pearson correlation coefficient; p – p-value; BF – Bayes factor.

| Group | Metric | Statistics | HR | HF-HRV |
| --- | --- | --- | --- | --- |
| **HC - Placebo** | **RF** | r | 0.13 | -0.49 |
|  |  | p | 0.67 | 0.07 |
|  |  | BF | 4.55 | 1.01 |
| **CHR-P - Placebo** | **RF** | r | 0.25 | -0.16 |
|  |  | p | 0.21 | 0.45 |
|  |  | BF | 3.04 | 4.96 |
| **HC - Oxytocin** | **RF** | r | -0.15 | 0.32 |
|  |  | p | 0.58 | 0.23 |
|  |  | BF | 4.55 | 2.56 |
| **CHR-P - Oxytocin** | **RF** | r | 0.24 | -0.39* |
|  |  | p | 0.23 | 0.04 |
|  |  | BF | 3.27 | 0.89 |

**Figure S4 – *Post-hoc* power analyses.** In this figure, we present the results of *post-hoc* power analyses, we conducted using *jpower* package of JAMOVI (1.2.17.0), to investigate what is the lowest effect size our samples would allow us to detect with an acceptable statistical power of 80% in two-tailed tests for each of our main hypothesis (panel A - Hypothesis 1: differences in HR and HRV between healthy and men at CHR-P under placebo (Independent samples T-test); panel B – Hypothesis 2: effects of intranasal oxytocin on HR and HRV in men at CHR-P (Paired samples T-test). In the upper row, we present contour power plots, which show how the sensitivity of the test changes with the hypothetical effect size and the sample size in the design. In the lower row, we present tables summarizing the range of effect sizes our samples would allow us to detect or miss with different degrees of certainty.


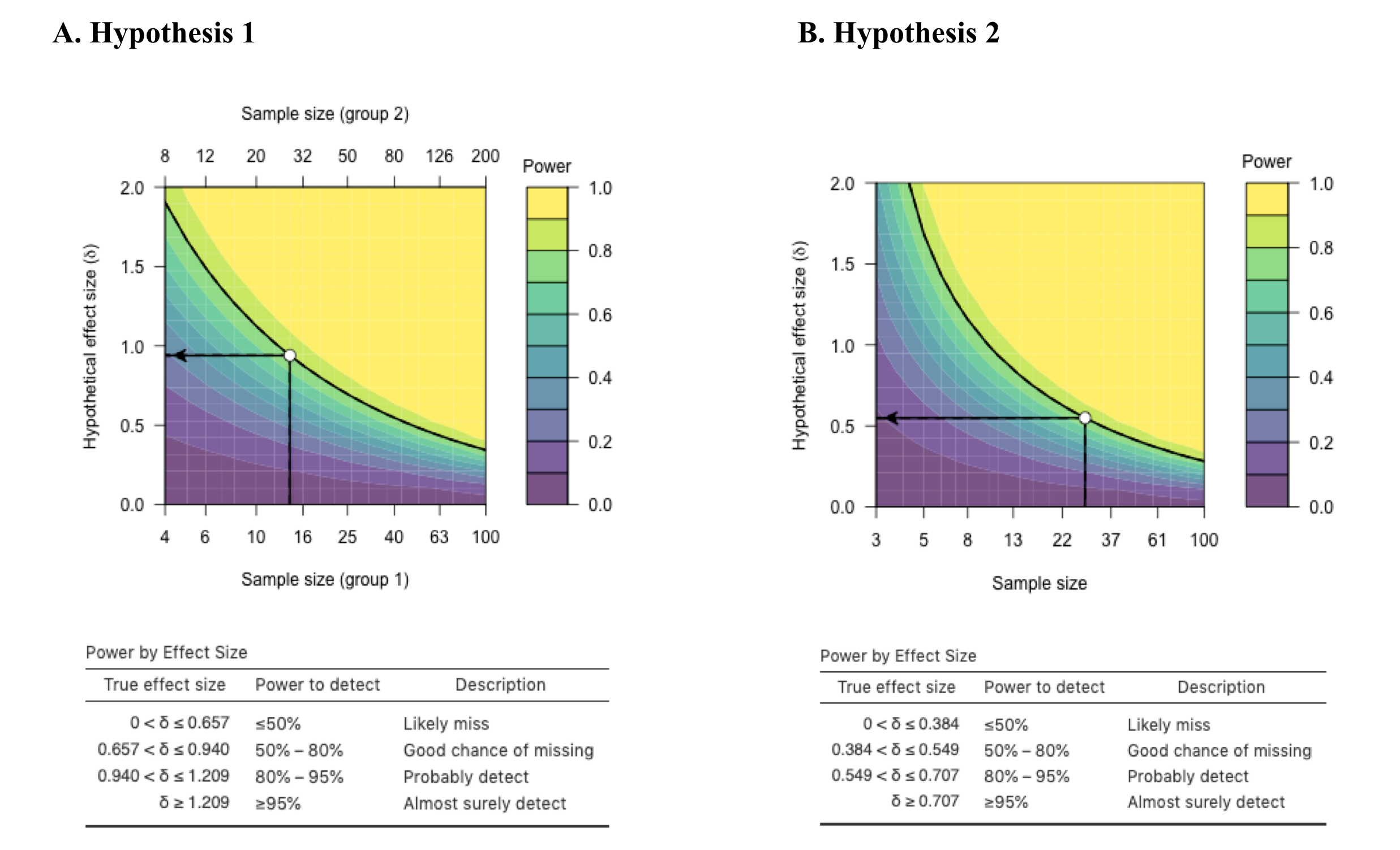


**Supplementary References**

1 Aysin, B. & Aysin, E. Effect of respiration in heart rate variability (HRV) analysis. *2006 28th Annual International Conference of the Ieee Engineering in Medicine and Biology Society, Vols 1-15*, 1726-+ (2006).

2 Shaffer, F. & Ginsberg, J. P. An Overview of Heart Rate variability Metrics and Norms. *Frontiers in Public Health* **5**, doi:UNSP 258

10.3389/fpubh.2017.00258 (2017).

3 Quintana, D. S. & Heathers, J. A. J. Considerations in the assessment of heart rate variability in biobehavioral research. *Frontiers in Psychology* **5**, doi:ARTN 805

10.3389/fpsyg.2014.00805 (2014).

4 Quintana, D. S. *et al.* Resting-state high-frequency heart rate variability is related to respiratory frequency in individuals with severe mental illness but not healthy controls. *Scientific Reports* **6**, doi:ARTN 37212

10.1038/srep37212 (2016).

5 Reardon, M. & Malik, M. Changes in heart rate variability with age. *Pace* **19**, 1863-1866, doi:DOI 10.1111/j.1540-8159.1996.tb03241.x (1996).

6 Bassi, D., Cabiddu, R. & Borghi-Silva, A. Body Mass Index May Influence Heart Rate Variability Reply. *Arq Bras Cardiol* **111**, 640-641 (2018).

7 Ikawa, M. *et al.* Effects of combination psychotropic drug treatment on heart rate variability in psychiatric patients. *Psychiatry and Clinical Neurosciences* **55**, 341-345, doi:DOI 10.1046/j.1440-1819.2001.00873.x (2001).
